# Cerebral Microbleeds During Transcatheter Aortic Valve Replacement: A Prospective Magnetic Resonance Imaging Cohort

**DOI:** 10.1161/CIRCULATIONAHA.121.057145

**Published:** 2022-06-20

**Authors:** Eric Van Belle, Nicolas Debry, Flavien Vincent, Grégory Kuchcinski, Charlotte Cordonnier, Antoine Rauch, Emmanuel Robin, Fanny Lassalle, François Pontana, Cédric Delhaye, Guillaume Schurtz, Emmanuelle JeanPierre, Natacha Rousse, Caterina Casari, Hugues Spillemaeker, Sina Porouchani, Thibault Pamart, Tom Denimal, Xavier Neiger, Basile Verdier, Laurent Puy, Alessandro Cosenza, Francis Juthier, Marjorie Richardson, Martin Bretzner, Jean Dallongeville, Julien Labreuche, Mikael Mazighi, Annabelle Dupont-Prado, Bart Staels, Peter J. Lenting, Sophie Susen

**Affiliations:** 1 Cardiology Department (E.V.B., N.D., F.V., C.D., G.S., H.S., S.P., T.P., T.D., X.N., B.V., M.R.), France.; 2 Department of Anesthesiology (E.R., F.P., M.B.), France.; 3 Cardiac Surgery Department (N.R., F.J.), France.; 4 Heart and Lung Institute, Neuroradiology Department (G.K.), France.; 5 Degenerative and Vascular Cognitive Disorders, Department of Neurology (C. Cordonnier, L.P.), France.; 6 Hematology and Transfusion Department (A.R., F.L., E.J., A.D.-P., S.S.), France.; 7 CHU Lille (J.L.), France.; 8 INSERM Unité 1011 (E.V.B., N.D., F.V., A.R., E.J., A.D.-P., B.S., S.S.), Univ. Lille, Inserm, CHU Lille, U1172 - LilNCog - Lille Neuroscience & Cognition, F-59000 Lille, France; 9 (C. Cordonnier, L.P.), Université Lille, France.; 10 EA 2694–Santé Publique: Épidémiologie et Qualité des Soins (J.L.), Université Lille, France.; 11 INSERM UMR_S 1176 (C. Casari, P.J.L.), Université Paris-Sud, Université Paris-Saclay, Le Kremlin-Bicêtre, France.; 12 INSERM Unité 1167 (J.D.), Institut Pasteur de Lille, France.; 13 Department of Neurology, Hôpital Laribosière, APHP-NORD (M.M.), Université de Paris, France.; 14 Department of Interventional Neuroradiology, Fondation Adolphe de Rothschild, FHU NeuroVasc, INSERM U 1148 (M.M.), Université de Paris, France.

**Keywords:** aortic valve stenosis, cerebral microbleeds, hemostasis, transcatheter aortic valve replacement, von Willebrand factor

## Abstract

Cerebral microbleeds (CMBs) have been observed in healthy elderly people undergoing systematic brain magnetic resonance imaging. The potential role of acute triggers on the appearance of CMBs remains unknown. We aimed to describe the incidence of new CMBs after transcatheter aortic valve replacement (TAVR) and to identify clinical and procedural factors associated with new CMBs including hemostatic measures and anticoagulation management.

**Methods:**

We evaluated a prospective cohort of patients with symptomatic aortic stenosis referred for TAVR for CMBs (METHYSTROKE [Identification of Epigenetic Risk Factors for Ischemic Complication During the TAVR Procedure in the Elderly]). Standardized neurologic assessment, brain magnetic resonance imaging, and analysis of hemostatic measures including von Willebrand factor were performed before and after TAVR. Numbers and location of microbleeds on preprocedural magnetic resonance imaging and of new microbleeds on postprocedural magnetic resonance imaging were reported by 2 independent neuroradiologists blinded to clinical data. Measures associated with new microbleeds and postprocedural outcome including neurologic functional outcome at 6 months were also examined.

**Results:**

A total of 84 patients (47% men, 80.9±5.7 years of age) were included. On preprocedural magnetic resonance imaging, 22 patients (26% [95% CI, 17%–37%]) had at least 1 microbleed. After TAVR, new microbleeds were observed in 19 (23% [95% CI, 14%–33%]) patients. The occurrence of new microbleeds was independent of the presence of microbleeds at baseline and of diffusion-weighted imaging hypersignals. In univariable analysis, a previous history of bleeding (*P*=0.01), a higher total dose of heparin (*P*=0.02), a prolonged procedure (*P*=0.03), absence of protamine reversion (*P*=0.04), higher final activated partial thromboplastin time (*P*=0.05), lower final von Willebrand factor high-molecular-weight:multimer ratio (*P*=0.007), and lower final closure time with adenosine–diphosphate (*P*=0.02) were associated with the occurrence of new postprocedural microbleeds. In multivariable analysis, a prolonged procedure (odds ratio, 1.22 [95% CI, 1.03–1.73] for every 5 minutes of fluoroscopy time; *P*=0.02) and postprocedural acquired von Willebrand factor defect (odds ratio, 1.42 [95% CI, 1.08–1.89] for every lower 0.1 unit of high-molecular-weight:multimer ratio; *P*=0.004) were independently associated with the occurrence of new postprocedural microbleeds. New CMBs were not associated with changes in neurologic functional outcome or quality of life at 6 months.

**Conclusions:**

One out of 4 patients undergoing TAVR has CMBs before the procedure and 1 out of 4 patients develops new CMBs. Procedural or antithrombotic management and persistence of acquired von Willebrand factor defect were associated with the occurrence of new CMBs.

**Registration:**

URL: https://www.clinicaltrials.gov; Unique identifier: NCT02972008.

**Clinical Perspective**

**What Is New?**

- We prospectively investigated the prevalence of cerebral microbleeds (CMBs) before and after transcatheter aortic valve replacement as well as procedural factors and outcomes associated with this occurrence in 84 patients.
- Approximately 1 patient of 4 has CMBs before transcatheter aortic valve replacement and nearly 1 of 4 develops new microbleeds after transcatheter aortic valve implantation.
- Anticoagulation management and an acquired von Willebrand factor multimer defect, in particular when this defect persists at the end of the procedure, are associated with the occurrence of new postprocedural CMBs.

**What Are the Clinical Implications?**

- A prolonged procedure increases exposure to anticoagulation and favors the appearance of new CMBs.
- The persistence of high shear-inducing defect in von Willebrand factor also promotes the appearance of CMBs.
- The pathophysiologic mechanisms of CMBs and their effect on long-term outcomes and especially on neurocognitive evolution require further study.

Cerebral microbleeds (CMBs), which consist of extravasation of blood components through fragile cerebral microvascular walls,^1,2^ are identified as small hypointense foci detected by T2*-weighted or susceptibility-weighted magnetic resonance imaging (MRI).^3–5^ They are common in patients with cerebral small vessel disease, which is present in 10% to 25% of people >70 years of age.^6,7^ CMBs appear progressively over time (≤7.0%/year)^7^ and are associated with increased risk for future ischemic strokes,^8^ cognitive impairment or dementia,^1^ and intracerebral hemorrhage, especially in the setting of antithrombotic use.^9^ Some risk factors for CMBs have been identified, such as aging, hypertension, and diabetes,^1,6,8^ but the underlying mechanisms leading to these hemorrhagic lesions remain elusive.^11^ Acute CMBs have been reported in patients with endocarditis,^12,13^ undergoing cardiac surgery,^14^ or receiving circulatory assist devices,^15,16^ all blood flow conditions associated with high shear stress.

von Willebrand factor (VWF) is a large multimeric glycoprotein involved in hemostasis,^17^ vascular structure,^18^ inflammation, and angiogenesis.^19^ Changes in blood flow and shear conditions may provoke the proteolytic degradation of the largest multimers, which are the most active in hemostasis.^18^ For instance, patients with severe heart valve disease (HVD), in particular elderly patients with aortic stenosis (AS), have an acquired VWF defect exposing them to an increased risk of mucosal and in particular gastrointestinal bleeding.^20^ This hemostasis disorder is corrected acutely by transcatheter aortic valve replacement (TAVR) in most patients, although it may persist in those with paravalvular regurgitation after incorrect placement of the valve.^21–23^ Recent preclinical studies have demonstrated higher prevalence of cerebral CMBs in animal models (SHRSP [spontaneously hypertensive stroke prone] rats) with high pulse pressure. The combination of VWF inactivation and high pulse pressure may trigger cerebral bleeding, suggesting that a VWF multimer defect could also increase the risk of CMBs in the setting of high pulse pressure.^24^

On the basis of these observations, we designed the prospective METHYSTROKE study (Identification of Epigenetic Risk Factors for Ischemic Complication During the TAVR Procedure in the Elderly; URL: https://www.clinicaltrials.gov; Unique identifier: NCT02972008) to investigate whether TAVR performed in patients with symptomatic AS and acquired VWF defect is associated with the occurrence of new CMBs. As part of this study, we attempted to assess whether patient or procedural measures, including antithrombotic regimen, and biological measures are associated with the occurrence of CMBs and to evaluate the association of CMBs with neurologic functional outcome.

## Methods

### Patient Selection

After the institutional review committee and ethics committee approved the study, all patients with AS undergoing TAVR at our institution who gave informed written consent were included in the prospective METHYSTROKE study, in which preprocedural and postprocedural cerebral MRIs were planned. As part of the screening process, patients were verified to have no contraindication for preprocedural MRI and to have a low probability of contraindication of postprocedural MRI. In that regard, patients with previous implantation of a pacemaker, who had signs of conduction abnormality at preprocedural (right bundle branch block) ECG, or who intended to undergo implantation of a self-expandable valve were excluded.

Participation included clinical data collection (including neurologic examination) and blood sampling (including analysis of VWF-related measures) during the procedure and follow-up.

**Figure 1.**
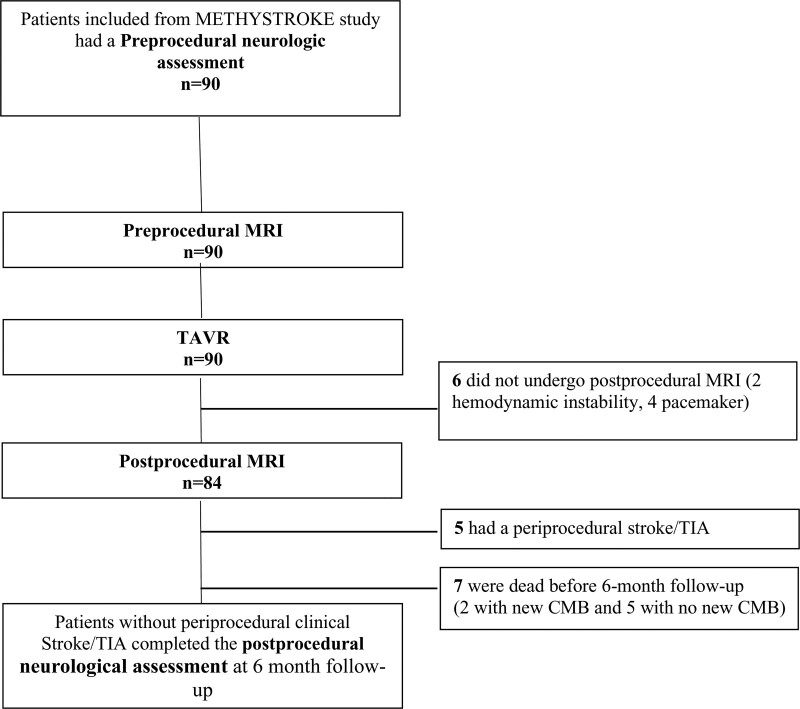
**Flowchart of clinical and imaging evaluations**. CMB indicates cerebral microbleed; MRI, magnetic resonance imaging; TAVR, transcatheter aortic valve implantation; and TIA, transient ischemic attack.

A flowchart of clinical and imaging evaluations is presented in Figure 1. The data supporting the findings of this study are available from the corresponding author and on reasonable request from qualified researchers trained in human subject confidentiality protocols.

### TAVR Procedure

Patients underwent transfemoral TAVR with a balloon-expandable Sapien valve (Edwards Lifesciences) because this device is less likely to require pacemaker implantation.^25^ All patients received aspirin before the procedure, with ongoing aspirin therapy after the procedure. Clopidogrel was not administered unless the patient was already receiving long-term clopidogrel treatment. No clopidogrel loading was performed.

Standard TAVR implantation techniques with unfractionated heparin anticoagulation (50 units/kg) with optional additional heparin administration depending on procedure duration or activated clotting time were followed as described previously.^23,26^ Protamine (1 mg for each 100 units of heparin) was administered at the end of the procedure at the discretion of the operator. Systolic blood pressure was maintained at >100 mm Hg throughout the procedure (except during rapid pacing). No embolic protection device was used during the procedure.

### Procedural Outcomes

All study patients were evaluated by 2 independent cardiologists according to the standardized Valve Academic Research Consortium–2^27^ recommendations, including device success, bleeding, acute kidney injury, access site complications, clinical transient ischemic attack (TIA) or stroke, death, new atrial fibrillation episode, and echocardiographic valve assessment.

### Per-Procedural VWF Multimer and Biology Analysis

Two distinct assays were performed to analyze the VWF multimer status: VWF multimer analysis through electrophoresis and through a functional whole blood assay (PFA-100; Siemens Healthcare Diagnostics). Both assays were performed on samples taken before (preprocedural) and 5 minutes after the end of the procedure (postprocedural) while the patient was still in the catheterization laboratory. VWF multimeric analysis was performed as previously described.^20,22,23^ The results were expressed as a ratio to normal pooled plasma (standard human plasma; Siemens Healthcare Diagnostics). High-molecular-weight (HMW) multimers defect was defined as reduced ratio of HMW multimers (>15-mer) present in plasma. With this method, the HMW:multimer ratio is defined as the number of HMW multimers in patient plasma sample divided by the number of HMW multimers in normal pooled plasma. The HMW:multimer ratio of normal pooled plasma is 1 (by definition) and a HMW multimers defect is defined as a reduced HMW:multimer ratio (less than 1).

Platelet count (×10^9^/L) and activated partial thromboplastin time (aPTT; in seconds) were measured before the procedure, at the time of valve implantation, and 5 minutes after the end of the procedure.

Closure time with adenosine diphosphate (CT-ADP) was assessed by PFA-100 using adenosine diphosphate cartridges (normal range, 68 to 121 seconds) as previously described.^20,22,23^

### Preprocedural and Postprocedural Cerebral MRI

Examination of the brain was performed on 1.5T MRI (Philips Ingenia; Philips Medical Systems) the day before and repeated 3 days after the TAVR procedure. The imaging protocol included the following sequences to identify CMBs and cerebral emboli: an axial diffusion-weighted imaging sequence (repetition time, 4600 ms; echo time, 100 ms; slice thickness, 4 mm; 2 diffusion gradient b values, 0 and 1000 s/mm^2^; field of view, 230 mm; matrix 105×224; acquisition time, 55 seconds), an axial T2*–gradient echo–weighted sequence (repetition time, 970 ms; echo time, 16 ms; flip angle, 18°; slice thickness, 4 mm; field of view, 230 mm; matrix 205×432; acquisition time, 2 minutes 40 seconds), an axial fluid-attenuated inversion recovery sequence (repetition time, 11 000 ms; echo time, 125 ms; inversion time, 2800 ms; slice thickness, 4 mm; field of view, 230 mm; matrix 264×512; acquisition time, 3 minutes 50 seconds), and an axial T1-weighted spin-echo sequence (repetition time, 6000 ms; echo time, 10 ms; slice thickness, 5 mm; field of view, 230 mm; matrix 205×432; acquisition time, 3 minutes 20 seconds).

**Figure 2.**
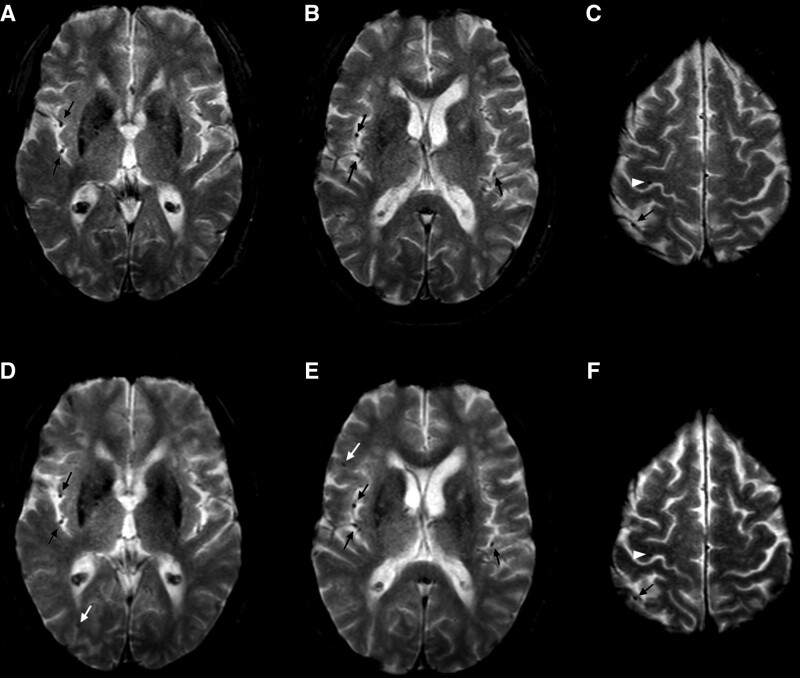
T2*-weighted gradient-echo sequence before and after transcatheter aortic valve implantation. Before (**A–C**) and after (**D–F**) transcatheter aortic valve implantation. This patient had a unique microbleed in the right precentral gyrus before transcatheter aortic valve implantation (white arrowhead; **C**). Two new microbleeds were observed after transcatheter aortic valve implantation, in the right frontal and temporal lobes (white arrows; **D** and **E**). Cerebral microbleeds were identified as homogeneous, round foci, <10 mm diameter, of low signal intensity. Flow void artifacts of the pial blood vessels (black arrows) were clearly distinguished from cerebral microbleeds by their location in the subarachnoid space and their tubular morphology on the adjacent slices. Preoperative and postoperative T2* images have been coregistered with an automated coregistration software dedicated to longitudinal comparison of magnetic resonance images using rigid transformations on the basis of mutual information (Longitudinal Brain Imaging; Philips Medical Systems).

According to recommendations^3,4^ and Standards for Reporting Vascular Changes on Neuroimaging criteria,^5^ CMBs were identified as homogeneous, round foci, 2 to 10 mm in diameter, of low signal intensity on the T2*–gradient echo sequence. The number, distribution, and size of CMBs were rated according to the Brain Observer Microbleed Scale.^3^ Low-signal lesions on the T2*–gradient echo sequence within a lesion compatible with an infarct were considered as hemorrhagic transformations rather than microbleeds and were excluded. Symmetrical foci of low signal intensity in the globus pallidus were considered as calcifications and were also excluded. Flow void artifacts of the pial blood vessels were distinguished from microbleeds by their morphology and correlation with T1 and fluid-attenuated inversion recovery images^1,8^ (Figure 2).

Incident CMBs were defined by the appearance of new microbleeds in a new location on the postprocedural MRI in comparison with the preprocedural MRI using automated coregistration software dedicated to longitudinal comparison of magnetic resonance images using rigid transformations on the basis of mutual information (LoBI; Philips Medical Systems).

Incident cerebral emboli (CE) were defined by the appearance of hyperintense lesions on postprocedural diffusion-weighted imaging in comparison with preprocedural diffusion-weighted imaging (i.e., that were not present on the preprocedural MRI).

All brain MRIs were rated by 2 independent neuroradiologists trained in the analysis of CMBs and blinded to clinical data. Validated qualitative and quantitative methods were applied (syngo.via version VA30; Siemens Healthcare). In case of disagreement, the final evaluation was obtained by consensus.

Interobserver kappa coefficients for the presence of CMBs and CE were 0.91 (95% CI, 0.83–0.98) and 0.92 (95% CI, 0.86–0.98), corresponding to very good agreement.

### Neurologic Examination

The neurologic trajectory covering neurologic deficits, cognition, disability, and quality of life was evaluated using a multimodal and repeated-testing approach in line with the recent Neurologic Academic Research Consortium recommendations for neurologic assessment for TAVR^28,29^ (Figure 1).

Neurologic and cognitive status was assessed by the same experienced neurologist before and 6 months after TAVR procedures following a standardized protocol as previously described in this population,^28^ including (1) the severity of the neurologic deficit, with the National Institutes of Health Stroke Scale (NIHSS); (2) the level of functional dependence, with the modified Rankin Scale (mRS; dependence was defined by a Rankin score >1); (3) cognition, with the Mini-Mental State Examination (MMSE); and (4) quality of life, with the EQ-5D.

### Study End Points and Analyses

The primary end point was the appearance of new CMBs on the postprocedural cerebral MRI. The secondary end point was the presence of microbleeds on preprocedural MRI.

Analyses included the identification of factors associated with the primary and secondary end points. The potential relationship between presence or incidence of cerebral emboli and the incidence of CMBs was investigated.

Additional analyses included evaluation of the consequences of the primary and secondary end points on early outcomes described by the standardized criteria proposed by the Valve Academic Research Consortium,^27^ on neurologic status evaluated at 6 months, and on 1-year mortality.

### Statistical Analysis

Quantitative variables are expressed as mean (SD) in the case of normal distribution or median (interquartile range) otherwise. Categorical variables are expressed as number (percentage). Normality of distributions was assessed using the Q-Q (quantile–quantile) plot and the Shapiro-Wilk test. We also report exact 95% CIs calculated by using the Clopper-Pearson method for primary and secondary end point rates.

Baseline and procedural characteristics were compared between patients with and without preprocedural new CMB, with and without postprocedural new CMB, and with and without postprocedural new CE by using the Fisher exact test for categorical variables or the Student *t* test (or the Mann-Whitney *U* test for non-Gaussian distribution) for quantitative variables. The magnitude of between-group differences was assessed by calculating standardized differences (estimated on rank-transformed data for non-Gaussian distribution); absolute values of 0.2, 0.5, and 0.8 were interpreted as small, medium, and large effect sizes.

Variables that were associated with postprocedural new CMBs with a *P*<0.05 in univariable analyses were entered into a multivariable logistic model using a Firth penalized likelihood approach and a forward stepwise selection on the basis of *P* values (*P*<0.05). Before developing the multivariable model, we examined the log-linearity assumption for quantitative variables (irrespective of results of previous univariable analyses) using restricted cubic spline functions as well as the absence of collinearity between candidate factors by calculating the variance inflation factors.^30^

We compared the 6-month change from baseline (6-month postprocedural versus postprocedural values) on MMSE and EQ-5D score between patients with and without postprocedural new CMBs by using analysis of covariance adjusted for baseline values; for MMSE, analysis of covariance was applied on rank-transformed values given the non-Gaussian distribution. Standardized differences derived from analysis of covariance models were reported as effect size using patients without postprocedural new CMBs as reference.

Statistical testing was done at the 2-tailed α level of 0.05. Statistical analysis was performed using the SAS software package, release 9.4 (SAS Institute).

## Results

### Patient Characteristics

Preprocedural MRI was performed in 90 patients with severe symptomatic AS undergoing implantation of the Sapien balloon-expandable valve through transfemoral access. Six patients could not undergo postprocedural cerebral MRI because of hemodynamic instability (n=2) or need for permanent pacemaker implantation after TAVR (n=4; Figure 1).

A total of 84 (93%) patients had both preprocedural and postprocedural MRI and constituted our study group (Figure 1). The baseline demographic, clinical, and echocardiographic characteristics of the study population are summarized in Table 1. Mean age was 80.6±5.6 years and 50% (n=42) were men. The mean logistic EuroSCORE was 20.0±4.3% and the predicted permanent stroke risk (Society of Thoracic Surgeons predicted risk of mortality) was 6.5±2.4%. Thirty-five percent (n=30) had a history of atrial fibrillation and 13% (n=11) had permanent atrial fibrillation. Seventy-one percent (n=60) had a history of hypertension and 21% (n=18) had previous cardiac surgery.

**Table 1.**
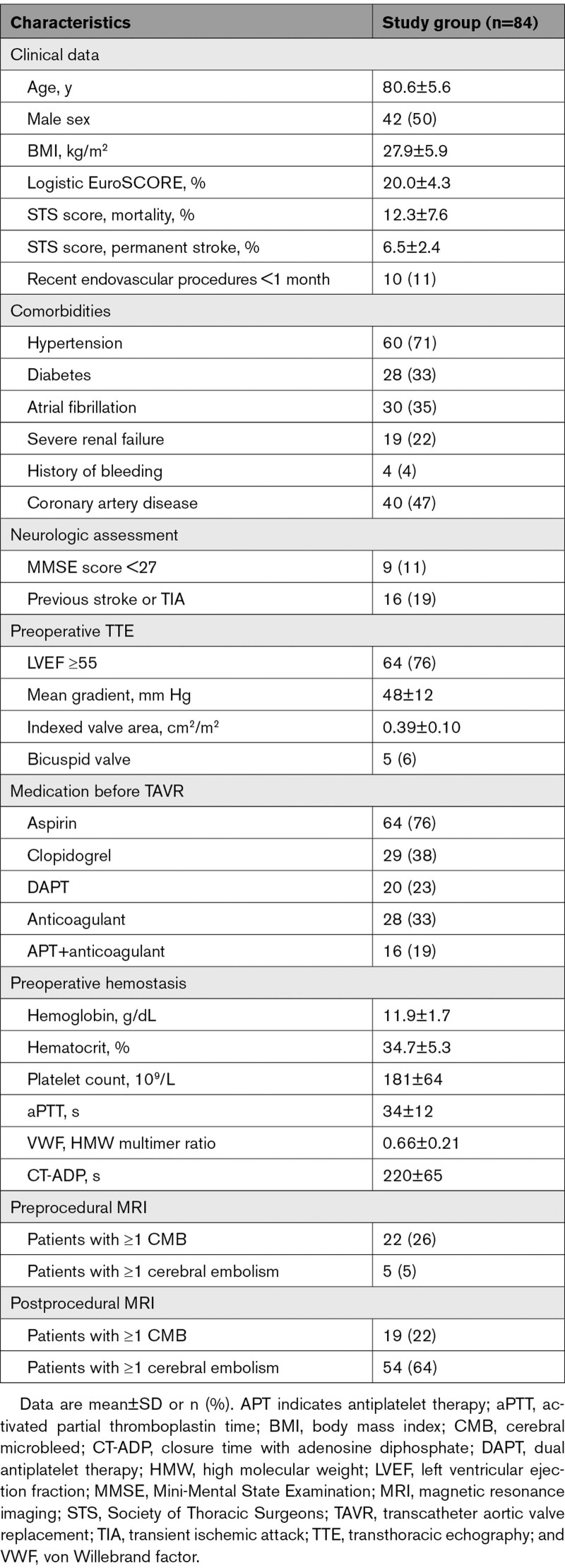
Baseline Demographic, Clinical, and Echocardiography Characteristics of the Study Population

The mean transaortic gradient was 48±13 mm Hg and the mean indexed valve area was 0.39±0.10 cm^2^/m^2^. A concomitant shear-induced acquired VWF defect was present in all patients as illustrated by a low mean HMW:multimer ratio (0.66±0.21) and a high mean CT-ADP (220±65 seconds).

### Device and Procedural Outcome

According to Valve Academic Research Consortium–2 criteria, device success was achieved in 84% (n=70) with no procedural mortality, no second valve, no moderate aortic regurgitation or greater, and no high transaortic gradient (20 mm Hg or more; Table S1). Five patients (5%) had clinical signs of TIA or stroke after TAVI; details about these patients are presented in Table 2.

**Table 2.**
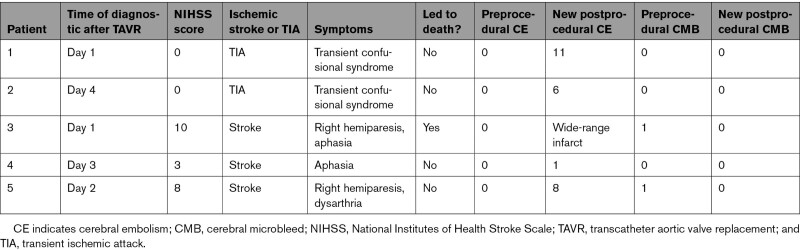
Details of Patients Presenting New Neurologic Deficits After TAVR Procedure (n=5)

Postimplantation transvalvular mean gradient decreased from 45.4±10.2 to 10.5±4.6 mm Hg (*P*<0.001) and the effective orifice area increased from 0.72±0.23 to 1.91±0.33 cm2 (*P*<0.001). Postimplantation HMW ratio increased from 0.66±0.21 to 1.03±0.26 (*P*<0.001) and CT-ADP decreased from 220±65 to 136±63 seconds (*P*<0.001).

Postprocedural aortic regurgitation grade 2 or greater was observed in 11% (n=9) by transthoracic echocardiography (TTE; Table S1). In each of these 9 patients, postprocedural VWF ratio was <0.8 (mean 0.73±0.06) and postprocedural CT-ADP was >180 seconds (mean 240±51 seconds).

Minor vascular complications involving femoral access occurred in 11% (n=10) of cases. There were no major vascular complications. Major bleeding (with no intracranial hemorrhage) was detected in 3.5% (n=3) of cases. There was 5% (n=4) new onset of atrial fibrillation (Table S1).

### Anticoagulation Management and Hemostatic Measures at the End of the Procedure

Protamine was used to reverse heparin at time of sheath removal in 23 patients (27%). During the procedure, peak aPTT was similar between patients who received protamine and those who did not (211±26 versus 214±23 seconds; *P*=0.65), but final aPTT was significantly lower in patients receiving protamine (65±34 versus 116±38 seconds; *P*=0.0003).

### Brain MRI Findings

Preprocedural MRI was performed at median time of 1 day (interquartile range, 1 to 3) before the procedure and the postprocedural MRI was performed 3 days (interquartile range, 2 to 4) after the procedure.

**Figure 3.**
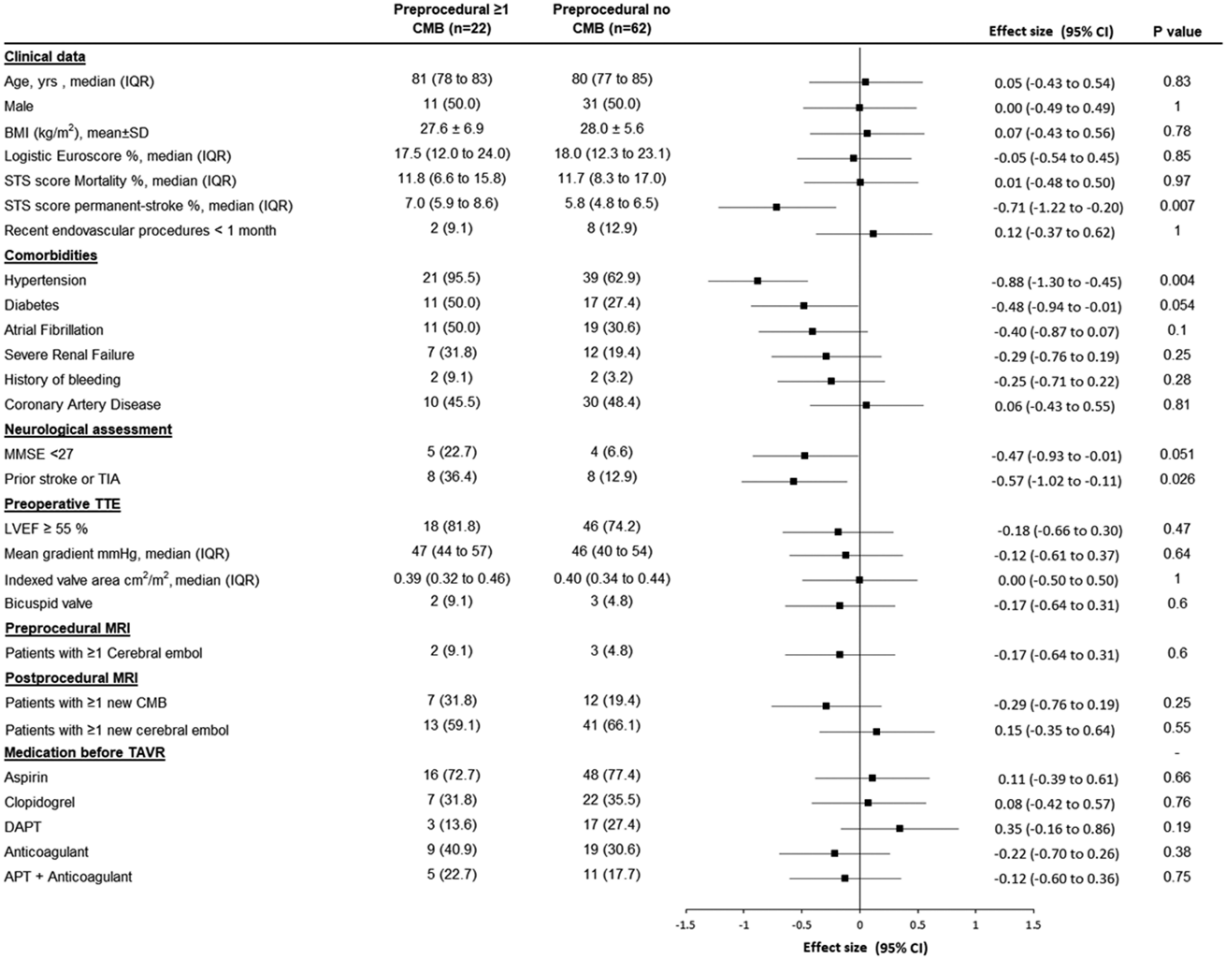
**Baseline factors associated with the presence of cerebral microbleeds on preprocedural magnetic resonance imaging.** Data are mean (SD), median (interquartile range [IQR]), or n (%) unless otherwise indicated. *P* values were calculated using the Fisher exact test for categorical variables or the Student *t* test (or the Mann-Whitney *U* test for non-Gaussian distribution) for quantitative variables. Effect sizes were the standardized differences (calculated on rank-transformed values for non-Gaussian quantitative variables); absolute values of 0.2, 0.5, and 0.8 were interpreted as small, medium, and large effect size. APT indicates antiplatelet therapy; BMI, body mass index; DAPT, dual antiplatelet therapy; LVEF, left ventricular ejection fraction; MMSE, Mini-Mental State Examination; MRI, magnetic resonance imaging; STS, Society of Thoracic Surgeons; TAVR, transcatheter aortic valve replacement; TIA, transient ischemic attack; and TTE, transthoracic echography.

Preprocedural brain MRI revealed at least 1 CMB in 22 patients (26% [95% CI, 17%–37%]) and at least 1 CE in 6% (n=5) of patients (Table 1, Figures 3 and 5, and Table S2). Preprocedural microbleeds were mostly identified in lobar regions (67%), deep regions (26%), or infratentorial regions (6%).

**Figure 4.**
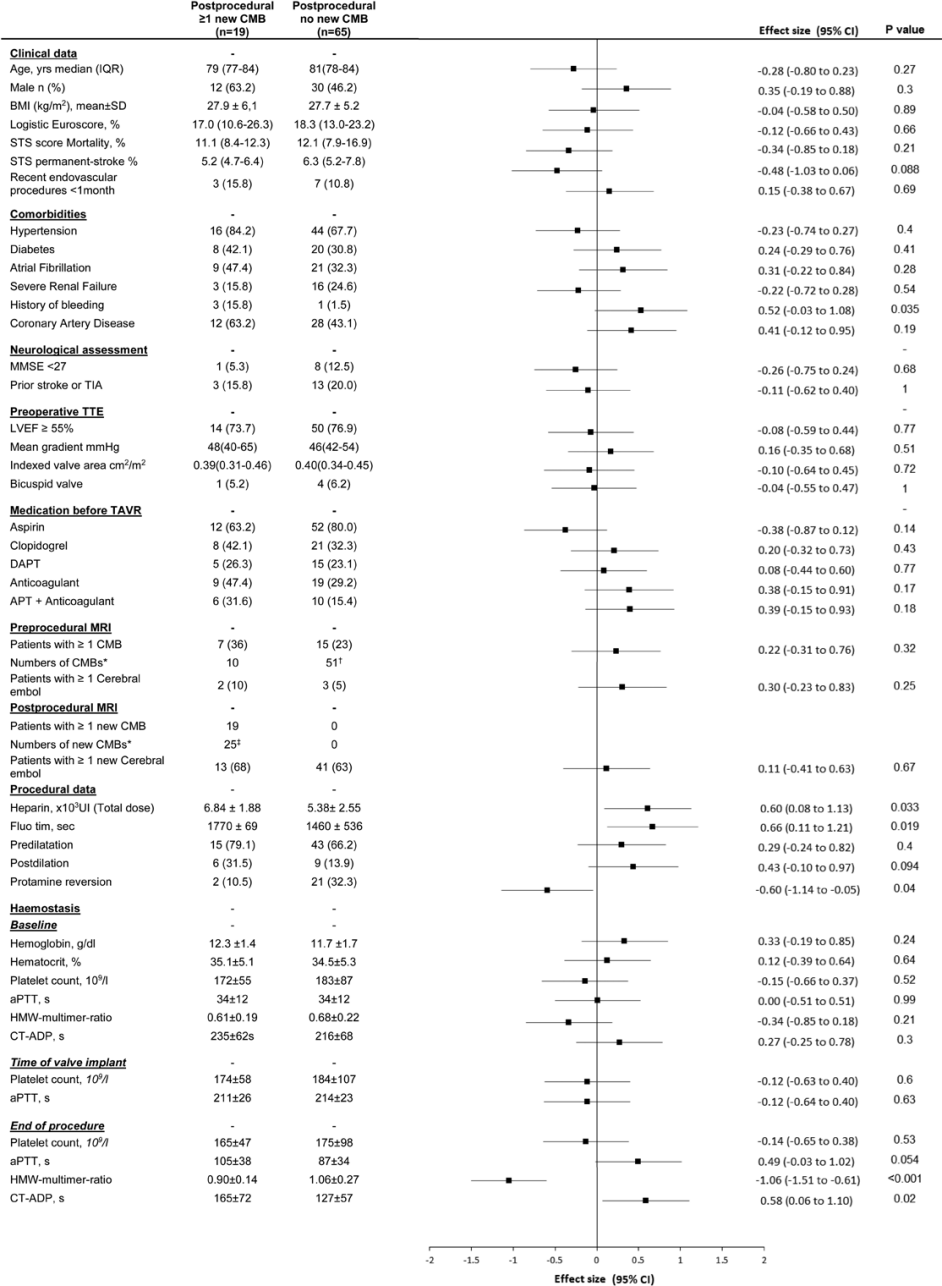
**Baseline and procedural factors associated with new cerebral microbleeds.** Data are mean (SD), median (interquartile range [IQR]), or n (%) unless otherwise indicated. New postprocedural cerebral microbleeds (CMBs) are CMBs present on postprocedural magnetic resonance imaging (MRI) that were not present on the preprocedural MRI. *P* values were calculated using the Fisher exact test for categorical variables or the Student *t* test (or the Mann-Whitney *U* test for non-Gaussian distribution) for quantitative variables. Effect sizes were the standardized differences (calculated on rank-transformed values for non-Gaussian quantitative variables); absolute values of 0.2, 0.5, and 0.8 were interpreted as small, medium, and large effect size. *Numbers of CMBs in the whole (sub)group. †One patient had 31 CMBs as part of a severe brain microangiopathy. ‡All 25 new CMBs were ≤5 mm. APT indicates antiplatelet therapy; aPTT, activated partial thromboplastin time; BMI, body mass index; CT-ADP, closure time with adenosine diphosphate (refers to platelet function analyzer); DAPT, dual antiplatelet therapy; HMW, high molecular weight; LVEF, left ventricular ejection fraction; MMSE, Mini-Mental State Examination; STS, Society of Thoracic Surgeons; TAVR, transcatheter aortic valve replacement; TIA, transient ischemic attack; and TTE, transthoracic echography.

Postprocedural brain MRI revealed at least 1 CMB in 34 patients (40%, 95% CI, 30%–52%) and at least 1 CE in 55 patients (65% [95% CI, 54%–75%]).

### Incident CMBs After TAVR

Between preprocedural and postprocedural MRI, 19 patients (23% [95% CI, 14%–33%]) had at least 1 new CMB (Table 1 and Figure 4) and 54 patients (64% [95% CI, 53%–74%]) had at least 1 new CE (Table S3). Only 15% (n=13) of patients had both new microbleed and new CE and 2.4% (n=2) of new microbleeds were associated with preprocedural CE. Seventy-one percent (n=60) of patients presented either new CMB or new CE.

Most of these new CMBs were unique (1 new microbleed [n=14], 2 microbleeds [n=4], 3 microbleeds [n=1]) and were identified in lobar regions (69%), deep regions (18%), or infratentorial regions (11%).

**Figure 5.**
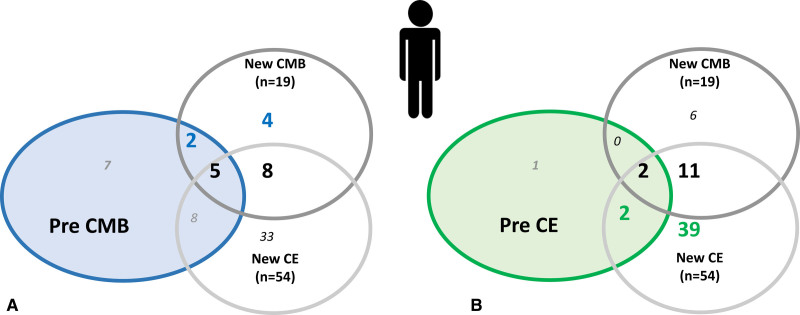
**Repartition of patients according to the presence of new CMB alone, new CE alone, or both on postprocedural MRI according to the presence of CMB or CE on preprocedural MRI.** Repartition of patients according to the presence of new cerebral microbleed (CMB) alone (n=6), new cerebral embol (CE) alone (n=41), or both (n=13) on postprocedural magnetic resonance imaging (MRI) according to the presence of CMB on preprocedural MRI (**A**) and the presence of CE on preprocedural MRI (**B**). Numbers in the colored circles represent the number of patients presenting with previous CMB (**A**) or CE (**B**) alone or associated with new CMB or new CE alone or in association. No relation was found between new postprocedural microbleed and preprocedural microbleed (*P*=0.23), preprocedural CE (*P*=0.33), or new postprocedural CE (*P*=0.88).

No statistical association was detected between CMBs and CE. No relation was found between new postprocedural microbleed and preprocedural CMB (*P*=0.23), preprocedural CE (*P*=0.33), or new postprocedural CE (*P*=0.88; Figures 4 and 5). Details about patients with CE on preprocedural MRI and patients with new CE on postprocedural MRI can be found in Tables S2 and S3. Of note, 59% (13/22) of patients with CMB on preprocedural MRI had a new CE on postprocedural MRI.

### Variables Associated With CMBs

Univariable associations with preprocedural CMBs are listed Figure 3 and included hypertension (*P*=0.004), diabetes (*P*=0.05), previous stroke or TIA (*P*=0.02), permanent stroke risk (Society of Thoracic Surgeons predicted risk of mortality) (*P*=0.007), and MMSE <27 (*P*=0.05).

Univariable associations with new postprocedural CMBs are listed in Figure 4 and included a previous history of bleeding (including gastrointestinal and cerebral bleeding; *P*=0.03) and procedural measures including valve postdilation (*P*=0.09) and a longer procedure (*P*=0.02).

A higher total dose of heparin was associated with a higher occurrence of new postprocedural CMBs (*P*=0.03). Protamine to reverse heparin at the end of the procedure was associated with a lower occurrence of new postprocedural microbleed (8% [n=2/23] versus 28% [n=17/61; *P*=0.03; Figure 4) with no increased occurrence of new CE (74% [17/23] vs 61% [37/61]; *P*=0.38). A lower final aPTT was also observed in patients without new postprocedural CMBs (87±34 versus 105±38 seconds; *P*=0.05).

Platelet counts before, during, or after the TAVR procedure (Figure 4) and variation of platelet count to peak (*P*=0.87) or to nadir (*P*=0.23) were not associated with the incidence of at least 1 new CMB on the postprocedural MRI.

A lower HMW:multimer ratio as measured immediately at the end of the procedure was associated with the occurrence of new CMBs detected on the MRI performed 3 days (interquartile range 2–4) after the procedure (0.90±0.14 versus 1.06±0.27; *P*=0.001). The same association was observed with higher CT-ADP (165±72 versus 127±57; *P*=0.02; Figure 4).

In forward stepwise multivariable analysis, including previous history of bleeding, total dose of heparin, fluoroscopy time, protamine reversion, HMW:multimer ratio at the end of the procedure, and CT-ADP level at end of the procedure, a prolonged procedure (odds ratio, 1.22 [1.03–1.73] for every 5 minutes of fluoroscopy time; *P*=0.02) and a postprocedural acquired VWF defect identified by a lower HMW:multimer ratio (odds ratio, 1.42 [1.08–1.89] for every lower 0.1 unit of the HMW:multimer ratio; *P*=0.004) were the only factors associated with the occurrence of new postprocedural CMBs.

### Cardiovascular Outcome and Relation to CMBs

We found no association between new postprocedural CMBs and in-hospital clinical outcomes, including the occurrence of periprocedural clinical stroke or TIA (*P*=0.21; Table S1). At 12 months, 5% of patients (n=1) with new CMBs and 12% (n=8) without new CMBs were deceased (*P*=0.25).

### Neurologic and Cognitive Status: Relation to CMBs

The analysis was conducted among the 73 patients without periprocedural clinical stroke or TIA and surviving at 6 months.

Baseline neurologic examination revealed that 12% of patients (n=9) had neurologic deficit (NIHSS 0 [n=1], 1 [n=6], 2 [n=2]; mRS 0 or 1 [n=3], 2 [n=4], 3 [n=2]). Patients with preprocedural CMB had a lower baseline MMSE (26±3 vs 28±1) and 6-month MMSE (26±3 vs 28±1) score than those without preprocedural CMB (both *P*=0.01). Presence of preprocedural CMB was not associated with altered functional capacities at baseline (mRS >1; n=2 [11.7%] versus n=4 [7.1%]; *P*=0.65] and at 6 months [mRS >1; n=2 [11.7%] versus n=3 [5.3%]; *P*=0.32).

**Figure 6.**
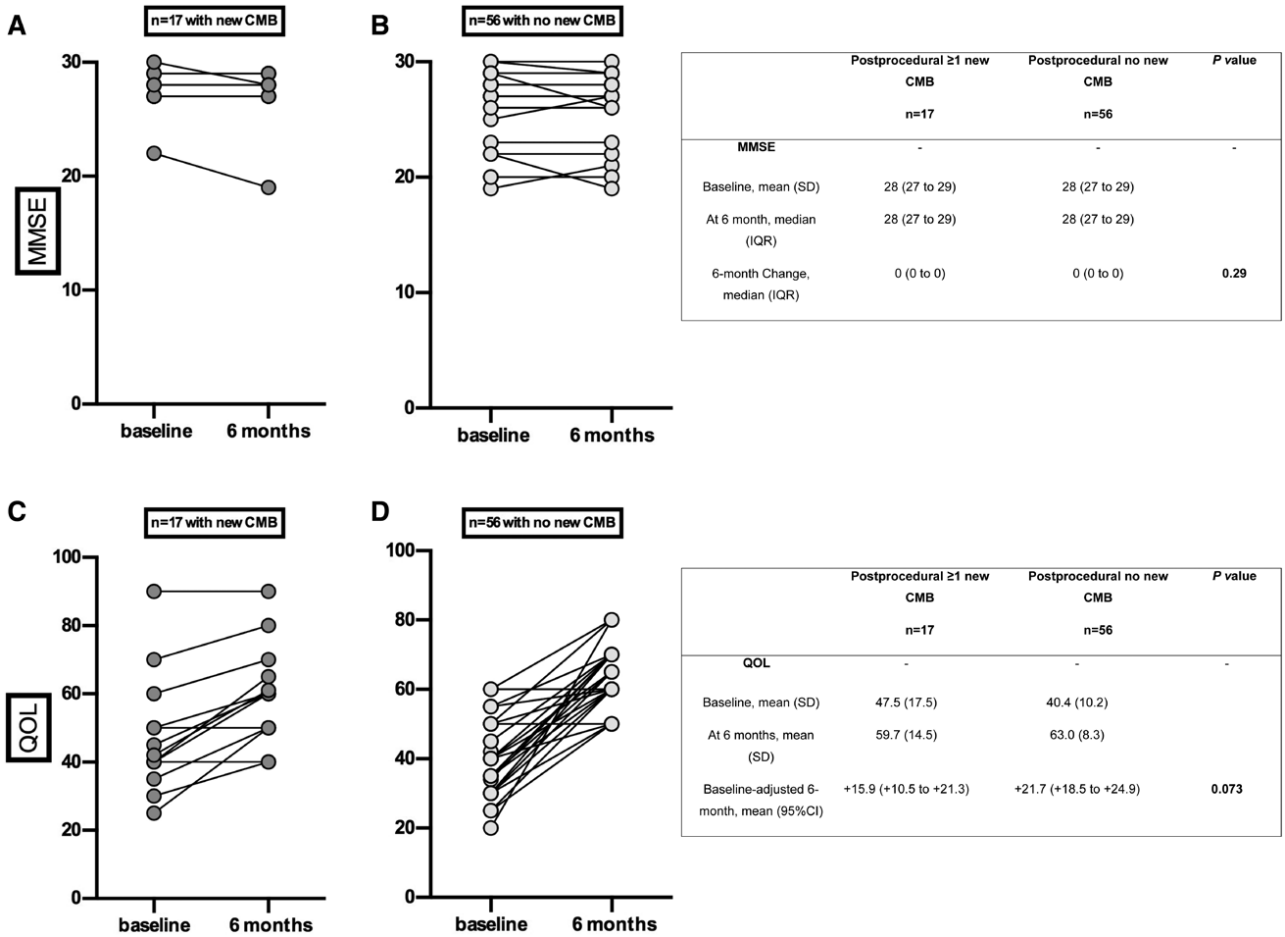
**Neurologic and quality of life evolution from before the procedure to 6-month follow-up according to the occurrence of new cerebral microbleeds after TAVR in the 73 patients without periprocedural stroke or TIA who were alive at 6 months**. Cognition assessed by Mini-Mental State Examination (MMSE) score at baseline and after 6 months in the presence (**A**) or absence (**B**) of new cerebral microbleeds (CMBs). Quality of life (QOL) assessed by EQ-5D score at baseline and after 6 months in the presence (**C**) or absence (**D**) of new CMBs. Patients with periprocedural stroke or transient ischemic attack (TIA) or who were deceased at 6 months were excluded. Baseline-adjusted effect sizes were standardized differences (calculated on rank-transformed values for MMSE). *P* value calculated using analysis of covariance on 6-month change adjusted for baseline values (calculated on rank-transformed values for MMSE).

At 6 months, an improvement in quality of life was observed (EQ-5D 42±12 versus 61±10; *P*<0.001), related to the success of the procedure without significant change in NIHSS, mRS, or MMSE score as compared with baseline. In particular, the occurrence of new postprocedural CMB did not affect functional capacities at 6 months (mRS >1, n=1 [5.9%] versus n=4 [7.1%]; *P*=0.78), MMSE score, or quality of life (Figure 6).

## Discussion

The current study including 84 patients with severe symptomatic AS undergoing transfemoral TAVR with preprocedural and postprocedural MRI performed 3 days apart demonstrates that (1) ≈1 patient out of 4 has CMBs before TAVR and nearly 1 out of 4 develops new CMBs after TAVR (23% [95% CI, 14%–33%]) and (2) longer fluoroscopy time and an acquired VWF multimer defect (in particular when this defect persists at the end of the procedure) are associated with the occurrence those new postprocedural CMBs.

### CMBs on Preprocedural MRI in Elderly Patients With Severe Symptomatic AS

In this population, the prevalence of CMBs (26% [95% CI, 17%–37%]) is similar to the reported prevalence of the general population at similar age^6,7^ (10%–25%). The clinical measures associated with the presence of preprocedural CMBs were also similar to those previously reported, such as hypertension (*P*=0.004), diabetes (*P*=0.05), and history of TIA or stroke (*P*=0.01), but not oral anticoagulation (*P*=0.43).^9,31^

### TAVR Procedures as a Trigger of New CMBs

To our knowledge, this study is the first to investigate the role of TAVR on the incidence of new CMBs. The key finding is that nearly 1 patient out of 4 experienced new CMBs (23% [95% CI, 14%–33%]).

The high incidence of new CMBs (23%) in such a short time window (3 days) contrasts with the low incidence observed over a 1-year period (4%–7%)^6,7^ in elderly patients with similar vascular risk factors receiving chronic antithrombotic medication and suggests that TAVR may trigger CMBs. Other cardiac interventions have been associated with the development of new CMBs (Table S4), but the association could not be established as only 1 MRI was performed in most cases (Table S4)^12,13,32,33^ or the time elapsed between the procedure and the second MRI was too long (>1 to 7 weeks).^14,34^

The current study also suggests that CMBs and emboli are independent events occurring in different patients (Figure 3) and that the occurrence of new postprocedural CMBs does not appear to be related to the presence of preprocedural CMBs (Figure 3). New CMBs and CE are not localized in the same territories and thus it is unlikely that new postprocedural CMBs represent hemorrhage within microinfarction areas.

### Role of Anticoagulation Regimen

The higher occurrence of new CMBs with a prolonged procedure or a prolonged exposure to anticoagulation during the procedure is consistent with the role of anticoagulation regimen previously demonstrated for “spontaneous” CMBs^35^ or for microbleeds associated with severe heart valve disease.^13,14^ Whereas the peak level of anticoagulation was not associated with development of CMBs, patients receiving a higher total dose of heparin were more likely to have new CMBs and those receiving protamine at the end of the procedure to reverse anticoagulation were less likely to have new CMBs (*P*=0.04). Patients with new CMBs had a longer aPTT value at the end of the procedure (*P*=0.05). This observation may extend the recently reported benefit of protamine on other bleeding complications after TAVR.^36^

### Acquired VWF Defect and CMBs

Acquired VWF defect in patients with heart valve disease has been associated with a persistent risk of bleeding, in particular gastrointestinal.^20^ However, its role in the occurrence of CMBs has never been investigated. Our study suggests that acquired VWF defect, in particular its persistence at the end of TAVR, may be associated with a higher risk of CMBs.

The observations that (1) all cases of CMBs reported to date in patients with acute cardiovascular disease are associated with a moderate (in patients with heart valve or prosthesis dysfunction; 567/608 patients [93%]; Table S4) or severe (in patients who received a ventricular assist device; 41/608 patients [7%]; Table S4) VWF multimer defect and (2) the clinical conditions with the most profound VWF multimer defect (assist device) were also those with the highest prevalence of CMBs (Table S4) are also highly supportive of a direct link between valve-related flow alterations, acquired VWF defect, and CMBs. In our study, CMBs were mostly located in lobar areas, similar to patients with heart valve disease.^13,14^

### Acquired VWF Defect Involvement in Blood–Brain Barrier Permeability?

Beyond the control of primary hemostasis, several studies have suggested that VWF regulates angiogenesis through multiple pathways,^37^ whereas VWF multimer defect promotes the appearance of angiodysplasia in the gastrointestinal tract and other arteriovenous malformations.^38^ VWF is also expressed abundantly in cerebral endothelial cells and could be involved in cerebral vessel remodelling.^18^ VWF also modifies and damages blood–brain barrier permeability in certain pathologic settings^39^ and might favor cerebral vessel wall rupture with consecutive microbleeds.^24^

### Hemodynamic Conditions and CMBs

The fundamental hemodynamic consequence of the acute correction of AS during TAVR is an acute shift from a narrowed pulse pressure to a normal one (or possibly a supranormal one in case of paravalvular regurgitation).^40^ This sudden hemodynamic change could trigger the appearance of microbleeds on background of the moderate to severe VWF multimer defect observed in patients with AS undergoing TAVR (or surgical aortic valve replacement^11,31^) or in patients with acute valve dysfunction.^9,10,29,30^ The report that CMBs are observed in animal models combining VWF defect with high blood pressure^24^ but not in those of VWF defect without high blood pressure^41^ are consistent with this hypothesis. The role of the sudden change in hemodynamic conditions likely explains why, despite the presence of a VWF multimer defect, the prevalence of CMBs in patients with AS before the procedure was not much higher than the one observed in a general population of the same age.^6^

### Paravalvular Regurgitation and CMBs

Previous works demonstrated that patients with postprocedural PVR after TAVR have persistent acquired VWF defect.^22,23^ In the current study, whereas we demonstrated a statistical relationship between persistent VWF defect and the occurrence of new CMBs (*P*=0.004), we did not demonstrate a statistical relationship between postprocedural PVR grade ≥2 identified on TTE and the occurrence of a new CMB (*P*=0.09). Collinearity between PVR and acquired VWF defect may explain part of this observation. This could also be favored by insufficient statistical power when investigating a link between 2 “dichotomous” events such as PVR and the occurrence of new CMBs. This is less of an issue when investigating the link with a “continuous” variable such as VWF defect. The use of TTE, which is less accurate than the gold standard transesophageal echocardiography (used in our previous works) to detect and grade PVR, may also decrease our ability to demonstrate this link.^22,23^ Three observations may support a role of the “modifiable factor” PVR as a trigger of the occurrence of new CMBs. First, patients with persistent PVR grade ≥2 on TTE had a 2.5-fold higher risk of new CMBs compared with those without (45% vs 20%; *P*=0.09). Second, the need for postdilation, mostly performed as an attempt to correct PVR, was also associated with a 2-fold higher risk of new CMBs (40% vs 18.8%; *P*=0.09). Third, our main observation that a persistent VWF defect, which has been shown to be primarily driven by PVR as identified by transesophageal echocardiography,^22,23^ triggers the appearance of new CMBs is by itself suggestive of a role of PVR on the appearance of VWF defect–mediated CMBs.

### Silent CMBs and Neurologic Trajectory After TAVR

Consistent with the notion that CMBs are considered silent lesions,^6,11^ we did not observe any focal deficit in patients with CMBs when examining them at the time of the MRI. There was no association between CMBs and immediate postprocedural outcomes or long-term all-cause mortality (1-year follow-up). However, we found a relationship between the presence of CMBs at baseline and moderate cognitive decline as measured by the MMSE.

In our population, and as previously reported,^42^ new CMBs per se did not worsen cognitive function at 6 months.

Whereas spontaneous CMBs, such as those observed on preprocedural MRIs, are known to predict long-term neurologic prognosis,^43^ the role of new periprocedural microbleeds is unclear. The small number of patients, the low number of new microbleeds per patient induced by the procedure, and the short follow-up may limit the ability of the current study to detect the role of these lesions on cognitive function at 6 months after TAVR. Additional studies involving more patients and longer follow-up are needed to clarify this issue.

### Study Limitations

This is a single-center study and results may have been influenced by local patient management. However, because the main purpose of the study was not to evaluate a specific device or a specific method of management but rather the natural history of CMBs during TAVR and their causes, this should not have affected our findings. Although our study is one of the largest MRI studies in TAVR, CMBs were analyzed on 1.5T MRI and the total number of patients with new CMBs was small and limits the strength of the multivariable analysis. This also limits the interpretation of the relationship between CMBs and neurologic outcomes.

Susceptibility-weighted imaging is more sensitive than T2* for microbleeds detection. In a cohort study of 246 patients with cognitive disorders, the use of susceptibility-weighted imaging modestly increased microbleeds prevalence from 17% to 21%.^44^ On the other hand, T2* is associated with a shorter acquisition time and is less prone to motion artifacts, making it more suitable for early postoperative assessment in an elderly population. In addition, susceptibility-weighted sequences are different according to vendors. The use of T2* allows comparisons of our results with those of previous studies.

### Conclusions

We report a high incidence (23% [95% CI, 14%–33%]) of new CMBs after TAVR. Anticoagulation management and persistence of acquired VWF defect were associated with development of new CMBs; the pathophysiologic mechanisms of CMBs and their role on long-term outcomes require further study.

## Article Information

### Acknowledgments

The authors thank Anais Gaul, Adeline Delsaux, and Marie Ghesquière, the research study nurses; the members of the catheterization laboratory; the cardiac operating room teams in the Department of Cardiology; and the hemostasis team in the national reference center for von Willebrand disease at Lille University Hospital.

### Sources of Funding

This work was supported by Lille-II University and by the National Research Agency (Programme d’Investissement d’Avenir) with the Hospital-University Research in Health program (Recherche Hospitalo-Universitaire, grant WILL-ASSIST HEART ANR-17-RHUS-0011).

### Disclosures

Dr Vincent has received a research grant from the Federation Française de Cardiologie and from Lille University Hospital. Dr Mazighi has received speaker honorary/advisory board compensation from Amgen, Boehringer Ingelheim, Air Liquide, and Acticor Biotech outside the current work. Dr Cordonnier served on a trial steering committee for Biogen, other for Boehringer Ingelheim and Pfizer, and as Associate Editor of *Stroke*. The other authors report no conflicts.

### Supplemental Material

Tables S1–S4

## Supplementary Material


