# Supplementary material for: Cerebral Microbleeds During Transcatheter Aortic Valve Replacement: A Prospective Magnetic Resonance Imaging Cohort
Source: Circulation. 2022 Jun 20;146(5):383–97. doi: 10.1161/CIRCULATIONAHA.121.057145 (PMC9345525; doi:10.1161/CIRCULATIONAHA.121.057145)
Supplement: Supplementary file 1 [file cir-146-383-s001.pdf]

CEREBRAL MICROBLEEDS DURING TAVR: A PROSPECTIVE MRI COHORT

Eric Van Belle MD-PhD*[1,2], Nicolas Debry MD*[1,2], Flavien Vincent MD[1,2], Grégory Kuchcinski MD[3], Charlotte Cordonnier MD-PhD[4], Antoine Rauch MD-PhD[2,5], Emmanuel Robin MD[6], Fanny Lassalle PharmD[5], François Pontana MD-PhD[6], Cédric Delhaye MD[1], Guillaume Schurtz MD[1], Emmanuelle JeanPierre PharmD [2,5], Natacha Rousse MD[7], Caterina, Casari PhD [8], Hugues Spillemaeker MD[1], Sina Porouchani MD[1], Thibault Pamart MD[1], Tom Denimal MD[1], Xavier Neiger MD[1], Basile Verdier MD[1], Laurent Puy[4], Alessandro Cosenza MD[1], Francis Juthier MD[7], Marjorie Richardson MD[1], Martin Bretzner MD[6], Jean Dallongeville MD-PhD [9], Julien Labreuche BST[10], Mikael Mazighi, MD-PhD[11], Annabelle Dupont-Prado PharmD-PhD[2,5], Bart Staels PhD[2], Peter J. Lenting PhD [8], Sophie Susen MD-PhD[2,5]

SUPPLEMENTAL MATERIAL

	Study group (n=84)	Postprocedural ≥1 new CMB (n=19)	Postprocedural no new CMB (n=65)	P value (Postprocedural)
VARC 2 criteria	-	-	-	-
Device success n (%)	70 (84)	15 (79)	55 (85)	0.73
All Bleedings n (%)	23 (27)	7 (36)	16 (24)	0.31
Vascular Complications n (%)	10 (11)	2 (10)	8 (12)	0.83
CHF n (%)	2 (2)	0 (0)	2 (3)	0.43
Stage 2-3 AKI n (%)	13 (15)	3 (15)	10 (15)	0.86
New AF episode n (%)	4 (5)	1 (5)	3 (5)	0.90
Clinical TIA/Stroke n (%)	5 (5)	0 (0)	5 (7)	0.21
IntraHospital death n (%)	1 (1)	1 (1)	0 (0)	0.06
TTE	-	-	-	-
Aortic Regurgitation ≥2	9 (11)	4 (21)	5 (8)	0.09
Mean gradient mmHg mean ± SD	10.5 ± 4.6	11.2± 4.2	10.4 ± 4.9	0.82
Aortic valve area cm^2 mean ± SD	1.77 ± 0.58	1.76 ± 0.50	1.77 ± 0.60	0.97

Supplemental Table 1. Early post-procedural outcome and new post-procedural CMB
Early post-procedural outcomes at 30 days (VARC-2 criteria).
AF: atrial fibrillation; AKI: acute kidney injury; CHF: congestive heart failure; CMB: Cerebral microbleed; TIA transient ischemic accident; TTE transthoracic echography.

	All (n=84)	Preprocedural ≥1 CE (n=5)	Preprocedural no CE (n=79)	P value
Clinical data	-	-	-	-
Age, yrs mean ± SD	80.6 ± 5.6	76.5 ± 5.6	80.9 ± 5.5	0.06
Male n (%)	42 (50)	3 (60)	39 (49)	0.64
BMI (kg/m^2) mean ± SD	27.9 ± 5.9	32.8 ± 11.1	27.6 ± 5.4	0.06
Logistic Euroscore % mean ± SD	20.0 ± 4.3	20.7 ± 7.2	20.0 ± 12.6	0.90
STS score Mortality % mean ± SD	12.3 ± 7.6	12.3 ± 5.7	12.4 ± 7.8	0.97
STS score permanent-stroke % mean ± SD	6.5 ± 2.4	7.9 ± 3.2	6.4 ± 2.4	0.17
Recent endovascular procedures < 1 month n (%)	10 (11)	4 (80)	6 (7)	**<0.001**
Comorbidities	-	-	-	-
Hypertension n (%)	60 (71)	3 (60)	57 (72)	0.55
Diabetes n (%)	28 (33)	4 (80)	24 (30)	**0.02***
Atrial Fibrillation n (%)	30 (35)	2 (40)	28 (35)	0.83
Severe Renal Failure n (%)	19 (22)	1 (20)	18 (22)	0.88
History of bleeding n (%)	4 (4)	0 (0)	4 (5)	0.60
Coronary Artery Disease n (%)	40 (47)	3 (60)	37 (46)	0.55
Neurological assessment	-	-	-	-
MMSE <27 n (%)	9 (11)	1 (20)	8 (10)	0.70
Prior stroke or TIA n (%)	16 (19)	3 (60)	13 (16)	**0.01**
Preoperative TTE	-	-	-	-
LVEF ≥ 55 n (%)	64 (76)	5 (100)	59 (74)	0.19
Mean gradient mmHg mean ± SD	48 ± 12	46.2 ± 15.3	48.4 ± 12.5	0.70
Indexed valve area cm^2/m^2 mean ± SD	0.39 ± 0.10	0.43 ± 0.10	0.39 ± 0.04	0.50
Bicuspid valve n (%)	5 (6)	1 (20)	4 (5)	0.17
Medication before TAVR	-	-	-	-
Aspirin n (%)	64 (76)	2 (40)	62 (78)	0.05
Clopidogrel n (%)	29 (38)	3 (60)	26 (32)	0.21
DAPT n (%)	20 (23)	0 (0)	20 (25)	0.19
Anticoagulant n (%)	28 (33)	1 (20)	27 (34)	0.51
APT + Anticoagulant n (%)	16 (19)	0 (0)	16 (20)	0.26
Preprocedural MRI	-	-	-	-
Patients with Microbleeds (CMB) n (%)	22 (26)	2 (40)	20 (25)	0.46
Patients with Cerebral emboli n (%)	5 (5)	NA	NA	NA
Postprocedural MRI	-	-	-	-
Patients with >= New Microbleeds (CMB) n (%)	19 (22)	2 (40)	17 (22)	0.33
Patients with New Cerebral emboli n (%)	54 (64)	4 (80)	50 (63)	0.44

Supplemental Table 2. Risk factors for the presence of CE on preprocedural MRI

APT: antiplatelet therapy; BMI: Body mass Index, CE: Cerebral emboli; CMB: Cerebral microbleed; DAPT: dual antiplatelet therapy; LVEF: left ventricular ejection fraction; MMSE: Mini-Mental State Examination; MRI: Magnetic Resonance Imaging; TIA: transient ischemic attack; TTE: transthoracic echography.

	Postprocedural ≥1 new CE(n=54)	Postprocedural no new CE (n=30)	Effect size (95% CI)	P value
**Clinical data**				
Age, yrs , median (IQR)	81 (77 to 85)	81 (78 to 84)	0.004 (-0.44 to 0.46)	0.99
Male	30 (55.6)	12 (40.0)	-0.32 (-0.75 to 0.12)	0.17
BMI (kg/m^2), mean±SD	28.0 ± 6.2	27.8 ± 5.3	-0.02 (-0.50 to 0.46)	0.93
Logistic Euroscore %, median (IQR)	18.5 (12.0 to 23.2)	17.0 (14.0 to 22.2)	0.00 (-0.47 to 0.47)	1
STS score Mortality %, median (IQR)	11.0 (7.6 to 16.9)	12.7 (8.8 to 17.0)	0.17 (-0.31 to 0.65)	0.49
STS score permanent-stroke %, median (IQR)	6.1 (5.0 to 7.5)	5.9 (5.0 to 6.8)	-0.03 (-0.51 to 0.45)	0.9
Recent endovascular procedures < 1 month	5 (9.3)	5 (16.7)	0.22 (-0.25 to 0.69)	0.32
**Comorbidities**				
Hypertension	36 (66.7)	24 (80.0)	0.31 (-0.16 to 0.77)	0.19
Diabetes	19 (35.2)	9 (30.0)	-0.11 (-0.55 to 0.33)	0.63
Atrial Fibrillation	19 (35.2)	11 (36.7)	0.03 (-0.42 to 0.48)	0.89
Severe Renal Failure	13 (24.1)	6 (20.0)	0.10 (-0.54 to 0.35)	0.67
History of bleeding	2 (3.7)	2 (6.7)	0.13 (-0.33 to 0.60)	0.61
Coronary Artery Disease	25 (46.3)	15 (50.0)	0.07 (-0.38 to 0.53)	0.74
**Neurological assessment**				
MMSE <27	5 (9.3)	4 (13.8)	0.14 (-0.32 to 0.60)	0.71
Prior stroke or TIA	11 (20.4)	5 (16.7)	-0.10 (-0.54 to 0.35)	0.68
**Preoperative TTE**				
LVEF ≥ 55 %	41 (75.9)	23 (76.7)	0.02 (-0.43 to 0.47)	0.94
Mean gradient mmHg, median (IQR)	47 (40 to 54)	46 (42 to 57)	0.02 (-0.43 to 0.47)	0.94
Indexed valve area cm^2/m^2, median (IQR)	0.40 (0.36 to 0.45)	0.39 (0.29 to 0.43)	-0.24 (-0.71 to 0.24)	0.31
Bicuspid valve	4 (7.4)	1 (3.3)	-0.18 (-0.61 to 0.25)	0.65
**Preprocedural MRI**				
Patients with ≥1 Cerebral embol	4 (7.4)	1 (3.3)	-0.18 (-0.61 to 0.25)	0.65
Patients with ≥1 CMB	13 (24.1)	9 (30.0)	0.13 (-0.32 to 0.59)	0.55
**Postprocedural MRI**				
Patients with ≥1 new CMB	13 (24.1)	6 (20.0)	-0.10 (-0.54 to 0.35)	0.67
**Medication before TAVR**				-
Aspirin	41 (75.9)	23 (76.7)	0.02 (-0.43 to 0.47)	0.94
Clopidogrel	16 (29.6)	13 (43.3)	0.29 (-0.18 to 0.75)	0.21
DAPT	10 (18.5)	10 (33.3)	0.34 (-0.12 to 0.81)	0.13
Anticoagulant	18 (33.3)	10 (33.3)	0.00 (-0.45 to 0.45)	1
APT + Anticoagulant	12 (22.2)	4 (13.3)	-0.23 (-0.66 to 0.20)	0.32

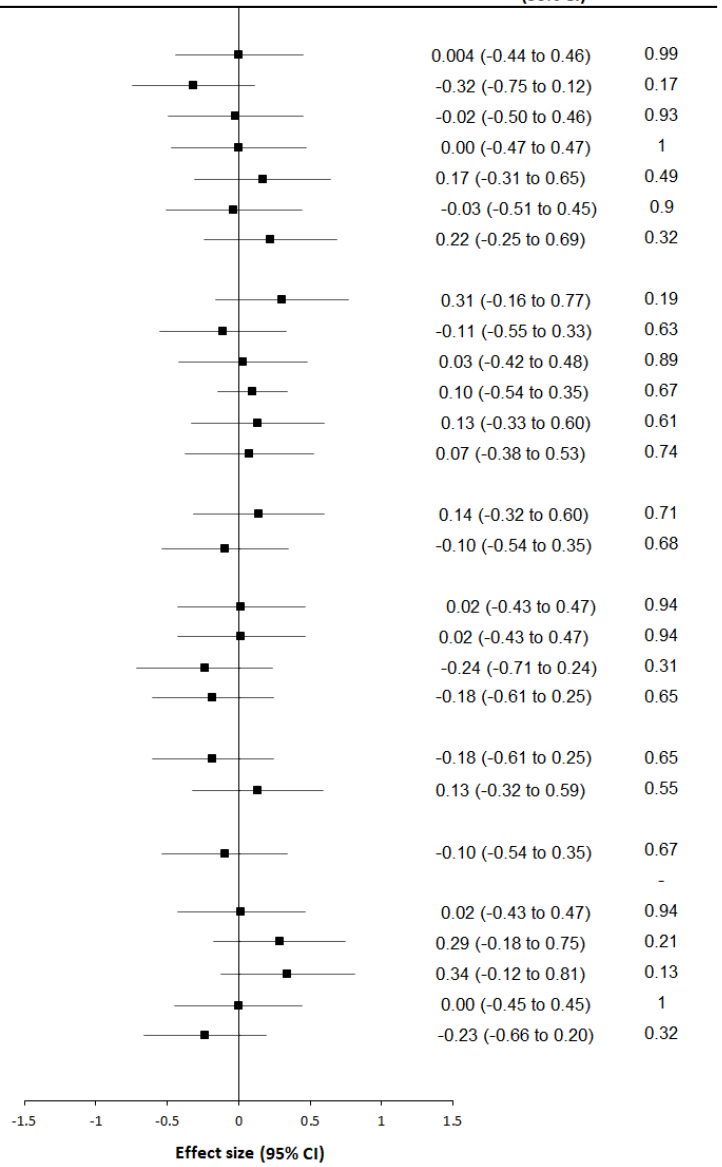

Supplemental Table 3. Risk factors for the presence of new CE on post-procedural MRI

APT: antiplatelet therapy; BMI: Body mass Index, CE: Cerebral embol; DAPT: dual antiplatelet therapy; LVEF: left ventricular ejection fraction; MMSE: Mini-Mental State Examination; TIA: transient ischemic attack; TTE: transthoracic echography.

	Index Event	N	Heart Valve disease	vWF defect*	Detailed data on vWF§	Detailed data On anticoagulation§	Pre-operative MRI	Post-operative MRI	Days between MRIs	Post-index event CMB rate	Pre-index event CMB rate	Localisation of CMB	Standardized Neurological evaluations
**Endocarditis**	-	-	-	-	-	-	-	-	-	-	-	-	-
**Duval et al. 2010**[31]	Endocarditis	130	YES	†	NO	NO	1.5T	-	NA	57%	-	-	-
**Goulenok et al. 2013**[9]	Endocarditis	30	YES	†	NO	NO	1.5T	-	NA	56%	-	-	-
**Iung et al. 2013**[10]	Endocarditis	120	YES	†	NO	NO	1.5T	-	NA	60%	-	-	-
**Hess et al. 2013**[32]	Endocarditis	109	YES	†	NO	NO	1.5T	-	NA	57%	-	85% lobar	-
**Total:**	-	**389**	-	-			-	-	-	-	-	-	-
**Cardiovascular Interventions**	-	-	-	-	-	-	-	-	-	-	-	-	-
**Sang-Beom Jeon et al. 2010**[33]	Cardiac valve Surgery CBP	19	YES	†	NO	NO	1.5T	1.5T	7 days	63%; 95%CI 38-83%	5% ; 95%CI 0.1-26%	46% lobar	-
**Liebeskind et al. 2012**[13]	ECLS in Children	6	NO	‡	NO	NO	-	1.5T	NA	100%	-	Most lobar	-
**Yoshioka et al. 2017**[12]	LVAD explantation	35	NO	‡	NO	Yes	-	3.0T	NA	**97%**	**-**	-	-
**Patel et al. 2019**[11]	Cardiac Surgery CBP	75	YES	†	NO	NO	1.5T	1.5T	7 weeks	76%	**36%**	70% lobar	YES
**Van Belle et al. 2022**	**TAVR**	**84**	**YES**	†	**Yes**	**Yes**	**1.5T**	**1.5T**	**3 days**	**23%; 95%CI 14-33%**	**26%, 95%CI=17-37%**	**69% lobar**	**YES**
**Total:**	-	**219**	-	-			-	-	-	-	-	-	-

Supplemental Table 4. Summary of studies describing cerebral microbleeds in acute cardiac scenarios

- : no data

CBP: cardiopulmonary bypass; CMB: Cerebral microbleed; CBP: Cardiopulmonary bypass; ECLS: extra-corporeal life support; LVAD: left ventricular assist device, MRI; Magnetic resonance imaging; NA: non applicable, T: Tesla (MRI); VWF: von Willebrand Factor.

*: according to published literature[20,22,45] all these clinical scenarios are known to be associated with at least moderate VWF multimer defect:

- † Moderate to severe VWF multimer defect is known to be present in patients with severe aortic stenosis and severe regurgitant valve disease[20,22]
- ‡ Severe to profound VWF multimer defect is known to be present in patients with assist device (ECLS or LVAD)[45]

§ : To describe whether these data were provided as part of the study.
